# Vagus nerve stimulation ameliorates cognitive impairment caused by hypoxia

**DOI:** 10.3389/fnbeh.2025.1555229

**Published:** 2025-06-06

**Authors:** Birendra Sharma, Krysten A. Jones, Laura K. Olsen, Raquel J. Moore, Frances S. Curtner, Candice N. Hatcher-Solis

**Affiliations:** ^1^Air Force Research Laboratory, Cognitive Neuroscience, 711th Human Performance Wing, Wright-Patterson AFB, OH, United States; ^2^Oak Ridge Institute for Science and Education, Oak Ridge, TN, United States; ^3^Integrative Health & Performance Sciences, UES, Inc., Blue Halo, Dayton, OH, United States; ^4^DCS Infoscitex, Dayton, OH, United States

**Keywords:** learning, memory, rats, vagus nerve stimulation, hypoxia, NGF, BDNF

## Abstract

**Introduction:**

Hypoxia significantly impairs cognitive function due to the brain’s high demand for oxygen. While emerging evidence suggests that vagus nerve stimulation (VNS) can enhance cognition, its effectiveness in mitigating behavioral and molecular impairments caused by hypoxia remains unknown. This study investigated whether VNS could alleviate hypoxia-induced deficits in cognitive performance and neurotrophin expression in rats.

**Methods:**

Healthy male Sprague–Dawley rats were randomly assigned to three groups: sham, hypoxia, and VNS + hypoxia. VNS was delivered during hypoxia (8% oxygen) exposure using 100 μs biphasic pulses at 30 Hz and 0.8 mA. Cognition and performance were assessed by behavioral testing and hippocampal tissue was collected for molecular analysis. NGF and BDNF mRNA levels were measured by quantitative PCR, and protein expression was evaluated by immunohistochemistry.

**Results:**

The passive avoidance test (PAT) performance was significantly reduced by hypoxia exposure compared to the sham group, and administration of VNS during hypoxia ameliorated this impairment. Hypoxia significantly reduced NGF and BDNF mRNA levels in the hippocampus 24 h post-exposure. VNS restored NGF mRNA to sham levels and partially increased BDNF mRNA. Immunohistochemistry results showed VNS significantly restored NGF protein expression in the hippocampus, while BDNF levels remained unchanged.

**Discussion:**

These findings suggest that VNS may serve as a promising intervention for cognitive impairments induced by hypoxia.

## 1 Introduction

Hypoxia, a condition characterized by insufficient oxygen supply to tissues, can result from environmental and pathological conditions such as high-altitude exposure, respiratory diseases, cardiovascular issues, and anemia (Adingupu et al., 2023; Iampietro et al., 2014; Kent et al., 2011; Grocott et al., 2007). The brain, requiring about 20% of the body’s oxygen supply, is particularly vulnerable to hypoxia. Oxygen deprivation in the brain can lead to various cognitive impairments, including memory loss, decreased attention span, slowed processing speed, and impaired executive functions (Wang et al., 2022; Bailey, 2019; Turner et al., 2015; Zhao et al., 2023). The severity of cognitive deficits often correlates with the duration and extent of oxygen deprivation, with acute conditions causing immediate cognitive impairments, while chronic hypoxia can lead to long-term cognitive decline (Bayer et al., 2011).

Hypoxia disrupts brain function through several mechanisms, including reduced ATP production, oxidative stress, and inflammation, all of which are well reported to impair cognitive function (Winn et al., 1981; Kawamura et al., 2019; Chen et al., 2014; Wilson et al., 2009; Mukandala et al., 2016). Reactive oxygen species (ROS) generated during hypoxia can damage neurons and the blood–brain barrier, further exacerbating cognitive deficits (Chen et al., 2014; Wilson et al., 2009). While the hypoxia-inducible factors (HIFs) can provide protection, they can also contribute to neuronal dysfunction and cognitive decline via different molecular pathways (Corcoran and O'Connor, 2013; Chen et al., 2020). Among these mechanisms, the disruption of neurotrophin regulation also plays a crucial role in diminishing cognitive resilience and compromising overall neural health in the brain (Rocco et al., 2018; Kumari et al., 2020).

Neurotrophins, such as brain-derived neurotrophic factor (BDNF) and nerve growth factor (NGF), play pivotal roles in supporting neuroplasticity, learning, memory, and cognitive resilience by supporting the survival, growth, and maintenance of cholinergic and noradrenergic neurons (Huang and Reichardt, 2001; Bathina and Das, 2015). Chronic stress and hypoxia can significantly reduce the expression of BDNF and NGF, which negatively impacts cognition and neuronal survival. For instance, prolonged immobilization stress lasting more than eight hours has been shown to significantly decrease BDNF and NGF mRNA levels in key hippocampal regions, including the CA1, CA3, and dentate gyrus (DG) (Ueyama et al., 1997). Similarly, exposure to three days of hypobaric hypoxia impairs cognitive performance and significantly reduces BDNF mRNA and protein expression in the same hippocampal regions (Kumari et al., 2020). The expression of NGF-induced early gene proteins, such as NGF1-A, is also significantly decreased in the CA1 region of the hippocampus after severe hypoxia exposure (Rybnikova et al., 2002). While these studies demonstrate neurotrophic changes, they primarily capture immediate effects and overlook potential alterations at later time points after exposure, which could be crucial for conditions like Acute Mountain Sickness (AMS).

AMS resulting from rapid ascent to high altitudes and reduced oxygen availability, typically appears within 4–24 h and can cause cognitive deficits lasting up to three days (Smedley and Grocott, 2013). Interestingly, cognitive impairments are most significant within the first 1–6 h of hypoxia exposure, even before AMS symptoms fully manifest [Banderet et al., 1986; Institute of Medicine (US), 1996]. While these immediate cognitive effects are well understood, the impact on cognitive performance and neurotrophins level beyond 24 h remains largely unexplored. This gap underscores the need for further research to determine whether the early cognitive changes persist or evolve after longer periods, such as 24 h following brief hypoxia exposure (4 h).

Given the profound impact of hypoxia on cognitive function and its negative effects on neurotrophin levels, developing targeted interventions is crucial. Current strategies focus on symptom alleviation and restoring oxygen supply but fall short of addressing neurotrophin dysregulation and the associated cognitive repercussions (Chen et al., 2023). Vagus nerve stimulation (VNS), an FDA approved neuromodulation technique, is a promising approach to address these limitations. VNS exerts widespread effects on brain function, including enhanced attention, memory consolidation, and plasticity, through stimulation of afferent vagal fibers that project to the nucleus tractus solitarius (NTS). This activates downstream neuromodulatory nuclei including the locus coeruleus (LC), dorsal raphe nucleus, and amygdala (Dorr and Debonnel, 2006; Beaumont et al., 2017; Collins et al., 2021). Activation of the LC leads to increased release of norepinephrine (NE) in the hippocampus and prefrontal cortex (Follesa et al., 2007). Norepinephrine activates *β*-adrenergic receptors, initiating the cAMP/PKA signaling cascade, which phosphorylates AMPA receptors, enhances calcium channel activity, and activates CREB-dependent transcription of plasticity-related genes such as BDNF and NGF (Chen et al., 2007; Hell et al., 1995). These molecular changes reinforce long-term potentiation and structural remodeling of synapses, ultimately enhancing learning and memory (Tully and Bolshakov, 2010; Izumi and Zorumski, 1999).

Preclinical VNS studies have demonstrated enhancements in recognition memory, spatial navigation, and fear extinction in rodent models, accompanied by synaptic remodeling and circuit-level plasticity (Liu et al., 2016; Jung et al., 2024; Peña et al., 2014; Souza et al., 2022; Alvarez-Dieppa et al., 2025). Clinical studies further support the cognitive benefits of VNS in conditions such as epilepsy, Alzheimer’s disease, and traumatic brain injury, with reported improvements in attention, working memory, verbal recall, and executive function (Clark et al., 1999; Sjögren et al., 2002; Ghacibeh et al., 2006; Sun et al., 2017). Moreover, both acute and chronic VNS have been shown to upregulate neurotrophins at the mRNA and protein levels, indicating its potential to induce both rapid and sustained neuroplastic changes (Olsen et al., 2022; Rosso et al., 2019; Sanders et al., 2019). The mechanisms by which short-term hypoxia affects neurotrophin expression and the extent to which VNS can reverse these effects remain unclear. This study investigated the impact of hypoxia on cognition and hippocampal neurotrophins in healthy male rats and examine whether VNS can ameliorate these changes through its neuromodulatory effects.

## 2 Materials and methods

### 2.1 Animals

**Figure 1:**
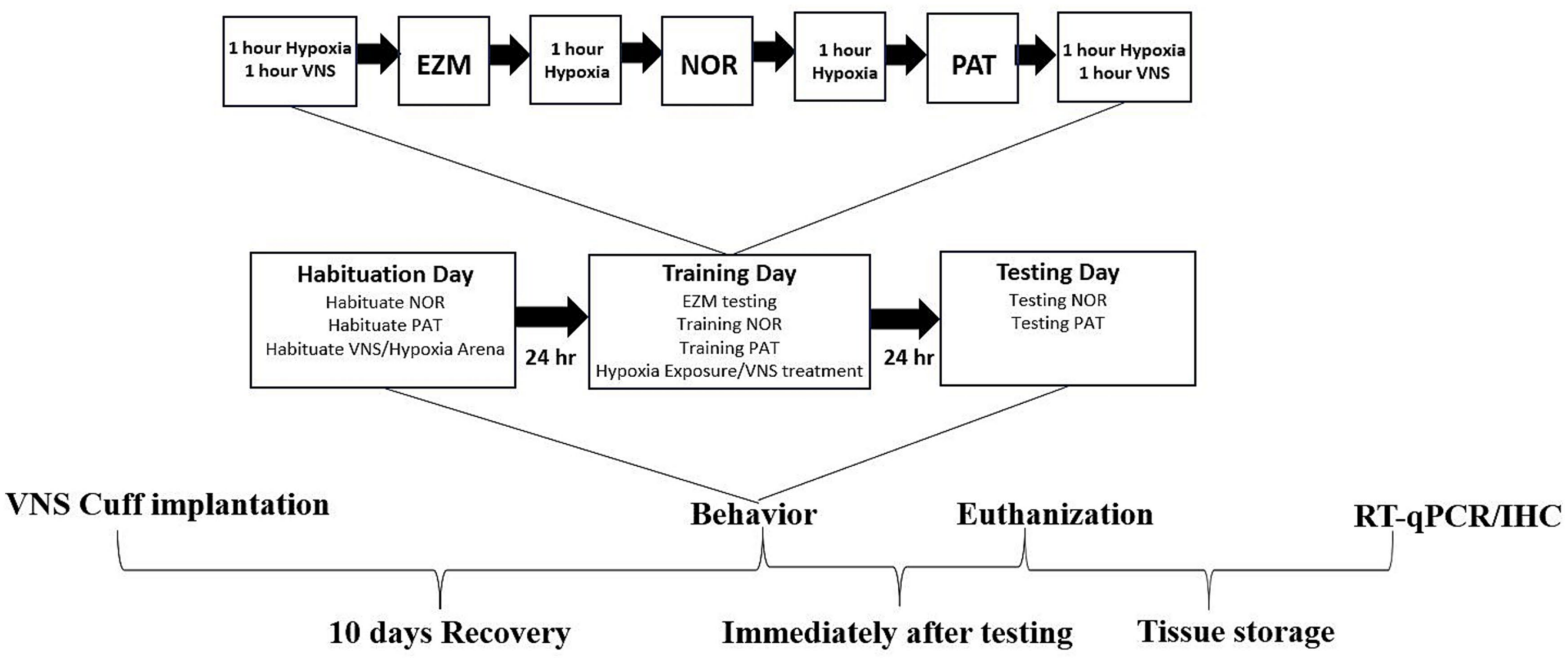
Experimental design. After recovery from surgical VN cuff implantation, all rats were utilized for behavioral, RT-qPCR and IHC analysis. Animals were habituated to the behavior and VNS/Hypoxia arenas on habituation day. The training day, 24 h after the habituation day, involved alternating periods of hypoxia exposure and VNS for a total of four hours of hypoxia and two hours of VNS, interspersed with behavior training and EZM testing. About 24 h after the training day, the rats were tested in the NOR and PAT paradigms. All animals were euthanized within two hours after completing behavior testing. Brain tissues were separated for left and right cerebral hemispheres and were either post-fixed in 4% PFA for IHC or dissected into different brain regions and stored in RNA-later for RT-qPCR.

The Wright-Patterson Air Force Base IACUC approved this study. All animal activities were conducted in an AAALAC accredited facility in compliance with all federal regulations governing the protection of animals and research, Department of Defense Instruction 3216.01, and the Guide for the Care and Use of Laboratory Animals. Male Sprague–Dawley rats (aged 5–7 weeks, Charles River) were group housed on a 12 h light cycle with *ad libitum* access to LabDiet 5,008 and water. Rats were allowed to acclimate for at least one week before any experimental procedures were conducted. Following acclimation, the rats underwent surgery for vagus nerve (VN) electrode cuff implantation on the left VN at the cervical level (see details below), followed by a recovery period of at least 10 days. Breathing reduction during delivery of 60 Hz, 0.8 mA constant current, 100 μs pulse stimulation for 30 s (Sanders et al., 2019) was determined and used to assign rats into three groups: sham, hypoxia, and VNS + hypoxia. *A priori* power analysis was conducted based on effect sizes demonstrating that VNS significantly enhances cognition and hippocampal neurotrophins level in rats (Olsen et al., 2022). Using those data, a minimum of *n =* 15 animals per group (behavioral outcomes) or 6 animals per group (molecular outcomes) would provide 80% power to detect statistically significant differences (*α* = 0.05). At 10–12 weeks of age, the rats underwent alternating periods of hypoxia exposure and VNS treatment for a total of 4 h of hypoxia exposure and 2 h of VNS, interspersed with behavior training (Figure 1). Approximately 24 h after the last VNS and hypoxia exposure, the rats were deeply euthanized and perfused intracardially with phosphate buffered saline (PBS). Half of the brain was post fixed in 4% paraformaldehyde (PFA) for 48 h at 4°C and then transferred to 30% sucrose for storage at 4°C. The other half of the brain was dissected into different regions, including hippocampus, and snap frozen in RNA later (AM7021; Thermo Fisher Scientific) for storage at −80°C. Reverse transcription quantitative polymerase chain reaction (RT-qPCR) and immunohistochemistry (IHC) were conducted to investigate the difference in BDNF and NGF expression resulting from hypoxia exposure or VNS intervention.

### 2.2 VNS cuff implantation surgery

Electrode wires composed of a platinum-iridium mix (90% platinum, 10% iridium) were affixed with gold connectors and linked to an Omnetics connector cap featuring four channels. Each wire, with a length of 2000 mm, was warmed and threaded through a catheter tube with a 0.08″ external diameter and a 0.04″ internal diameter. The electrical resistance of the electrode cuff was evaluated prior to its surgical insertion. Under the influence of isoflurane (5% for initiation, 2–3% for sustained sedation), an electrode cuff was surgically implanted around the VN and attached to a cranial cap. The head cap was anchored to the cranium with dental adhesive and screws made of stainless steel, incorporating the Omnetics four-channel setup. A subcutaneous path was established from the cranium to the left side of the neck to connect the electrode wires and the head cap. The left VN was gently unsheathed and separated from the adjacent carotid artery with the use of a glass nerve hook. The VN was then positioned within the catheter tube, ensuring direct engagement with the electrodes. The efficacy of the VNS cuff implantation was verified by determining a reduction in respiratory rate upon administering a continuous 60 Hz current of 0.8 mA with a pulse duration of 100 ms over a 30 s period.

### 2.3 Hypoxia exposure

A gas cylinder containing a mixture of 92% nitrogen and 8% oxygen was connected to a Plexiglas chamber measuring 40 cm x 44 cm x 37 cm or 48 cm x 25 cm x 20 cm through plastic tubing. The flow of the gas mixture into the chamber was regulated to maintain a specific hypoxia environment with 8% oxygen. The oxygen content inside the chamber was monitored using NeuLog logger sensors (E-2 version 3.0.5), with the readings displayed on a connected screen. The oxygen level within the chamber was logged every 15 min, ensuring the stability of the hypoxic conditions. The respiratory rates of the rats were checked every 30 min to monitor their response to the hypoxic environment. Sham animals were kept in the same chambers during hypoxia exposure but were exposed to ambient air conditions (~20% oxygen level).

### 2.4 Vagus nerve stimulation (VNS)

For rats undergoing VNS treatment during hypoxia exposure, the A365 Isostimulator from WPI was employed to deliver VNS on the left VN. The procedure comprised 15 sequences of biphasic pulse trains, each lasting 100 μs at a frequency of 30 Hz, with a steady 0.8 mA current, repeated every 18 s (Olsen et al., 2022) over a span of 60 min. During the stimulation, the rats had the liberty to roam in a plexy glass chamber measuring 40 cm x 44 cm x 37 cm. The scheduling of the pulse sequences was managed through a Micro3 1,401 CED device in conjunction with Signal 7 software. The sham and hypoxia rats were connected to the isostimulators but did not receive VNS.

### 2.5 Elevated zero maze (EZM)

The EZM was performed to evaluate anxiety-like behavior (Tucker et al., 2017). During the training session, rats were placed in an arena that was two feet high off the ground for a duration of five min. The arena was designed with two open arms opposite each other and two closed arms opposite each other. To ensure minimal bias, rats were placed at the center of a closed arm to begin, and the choice of starting arm (right or left closed arm) was randomized for each test. The rat’s movements and the amount of time spent in each arm were tracked and measured using Ethovision XT (version 11.5, Noldus).

### 2.6 Novel objection recognition (NOR)

The NOR test was performed to evaluate recognition memory (Olsen et al., 2022). On the habituation day, rats were placed in a NOR arena measuring 60 cm x 60 cm x 38 cm for a duration of five min. About 24 h later, on the training day, two of the same objects were introduced, each approximately 8 cm in radius and 10 cm in height, for a period of three min. The objects were crafted from glass to resemble a crown and spray-painted black. Following another 24 h period, on the testing day, rats were introduced to one familiar object (the same as on the training day) and one novel object. The novel object, designed to resemble a rook, matched the familiar object in size and was also spray painted black. On both the training and testing days, rats were allowed two min to acclimate to the arena before introducing the objects (positioned about 47 cm apart at opposite ends of the arena). Rats that failed to explore the objects for at least 15 s during the training phase were excluded from all NOR analysis. Ethovision XT system (v11.5) was used to track object exploration activity. The Novel Object Preference (NP) ratio was calculated with the formula:


$$\mathrm{NP}=\frac{\left(N\;\text{novel}–N\;\text{fimiliar}\right)}{\left(N\;\text{novel}+N\;\text{familiar}\right)}$$

### 2.7 Passive avoidance test (PAT)

The PAT was conducted to evaluate associative aversion learning and memory (Olsen et al., 2022). During the habituation phase, rats were placed in a 25 cm x 21 cm x 17 cm brightly lit arena connected to a dark arena of identical size via a gate (Gemini Avoidance System, San Diego Instruments Inc.). The gate was opened, and rats were allowed five min to explore both areas freely. Roughly 24 h later, on the training day, the rats were placed in the lit arena. After the gate opened, rats that entered the dark arena triggered the gate to close and a single 0.75 mA foot shock (FS) was administered in the dark arena. Following the mild FS, the rat spent an additional 30 s in the dark arena. The following day (testing day), the rat was placed in the brightly lit box and the latency to cross to the dark arena was measured. If the rat did not cross to the dark arena prior to the test cutoff time (900 s), the maximum time to cross was recorded. Rats that did not enter the dark arena and receive a FS during the training phase were excluded from the PAT analysis.

### 2.8 Reverse transcription quantitative polymerase chain reaction (RT-qPCR)

Hippocampus tissue stored in RNAlater was homogenized with 20 μL of Lysis Buffer from the MagMAX mirVana Total RNA Isolation Kit (A27828, Applied Biosystems) per 10 mg of tissue using a tissue lyser (TissueLyser II; Qiagen) and Tungsten Carbide Beads (2,060,816, Qiagen) Total RNA was then isolated from the lysate using the MagMAX mirVana Total RNA Isolation Kit following the manufacturer’s protocol. The quality of the isolated RNA was assessed spectrophotometrically with a Nanodrop ND-1000. The isolated RNA was reverse transcribed into cDNA with oligo-dT and RT reverse transcriptase at 37°C for 1 h in a total reaction volume of 20 μL using a High-Capacity RNA-to-cDNA Kit (4,387,406, Applied Biosystems). The qPCR reaction was performed on a StepOnePlus Real-Time PCR System (Applied Biosystems). Reactions contained 5ul of SYBR Green Master Mix (A25742, Applied Biosystems), 1 μL of each primer (8 mM concentration), 1 μL cDNA template, and 2 μL of ultrapure water to a reaction volume of 10 μL. To ensure accuracy, three technical replicates were performed for each rat biological sample and samples were only analyzed that passed quality control (StepOne Software v2.3). The following primers were used:

**Table 1:** 

NGF	Forward: 5’ AAGGGAGCGCATCGAGTTT 3’
	Reverse: 5’ CCTTTATTGCGCCCAGACACT 3
BDNF	Forward: 5’ TAAGAGTCTAGAACCTTGGGGAC 3’
	Reverse: 5’ TGGTGGAACTTTTTCAGTCACTA 3’
HRPT1	Forward: 5’GACCAGTCAACGGGGGACAT 3’
	Reverse: 5’ GGGGCTGTACTGCTTGACCA 3’

### 2.9 Immunohistochemistry (IHC) staining

Fixed brain tissue was sliced using a microtome (SM2010R, Leica Biosystem) to produce 30 μm sections from the hippocampal region for IHC staining of brain derived neurotrophic factor (BDNF) or nerve growth protein (NGF). The tissue was washed five times in PBS for five min and then blocked (PBS, 2% goat serum, 0.2% Triton X-100) for 1 h at room temperature. The tissue was then incubated overnight with rabbit anti-BDNF (1:250, sc-20981, Santa Cruz) or rabbit anti-NGF (1:200, sc-32300, Santa Cruz) at 4°C. The next day mouse anti-NeuN (1:2500, MAB377, Sigma) was added to the tissue samples and incubated for 2 h at room temperature. Tissues were then washed five times with PBS and subsequently incubated with fluorescent secondary antibodies (Alexa Fluor 488 goat anti-rabbit IgG and Alexa Fluor 594 goat anti-mouse, 111–545-144 and 115–585-146, Jackson ImmunoResearch Laboratories) for 1 h at room temperature. After five PBS washes and a phosphate buffer wash, the tissue was mounted, and cover slipped using Fluoroshield with DAPI (F6057, Sigma). Fluorescent images were obtained using an Olympus microscope at 20x magnification and analyzed via Image J and QuPath. NeuN staining was used to identify the pyramidal region of CA1 and CA2, stratum radiatum region of CA3, and subgranular zone of DG regions in the hippocampus. Quantification of BDNF or NGF positive cells were normalized to DAPI counts and were performed by a blinded experimenter for three technical replicates per rat sample.

### 2.10 Statistical analysis

Data from behavior testing and molecular analysis were examined for normality and homogeneity of variance using appropriate tests. Parametric behavioral data from the EZM and NP from the NOR test, RT-qPCR, and IHC were analyzed using ordinary one-way Analysis of Variance (ANOVA). Object exploration time and frequency of object exploration from the NOR test were analyzed using two way-mixed ANOVA. The Bonferroni *post hoc* test was conducted after one-way and two-way ANOVA. Non-parametric data from the PAT was analyzed using a Kruskal-Wallis test and Dunn post hoc test. No outliers were excluded from the analysis; all individual data points were retained to preserve transparency and reflect biological variability. All data are represented as means ± standard error of the mean (SEM). A *p*-value of less than 0.05 was considered significantly different. Detailed statistical analysis results are available for all analyses in Supplementary Tables 1–8.

## 3 Results

**Figure 2:**
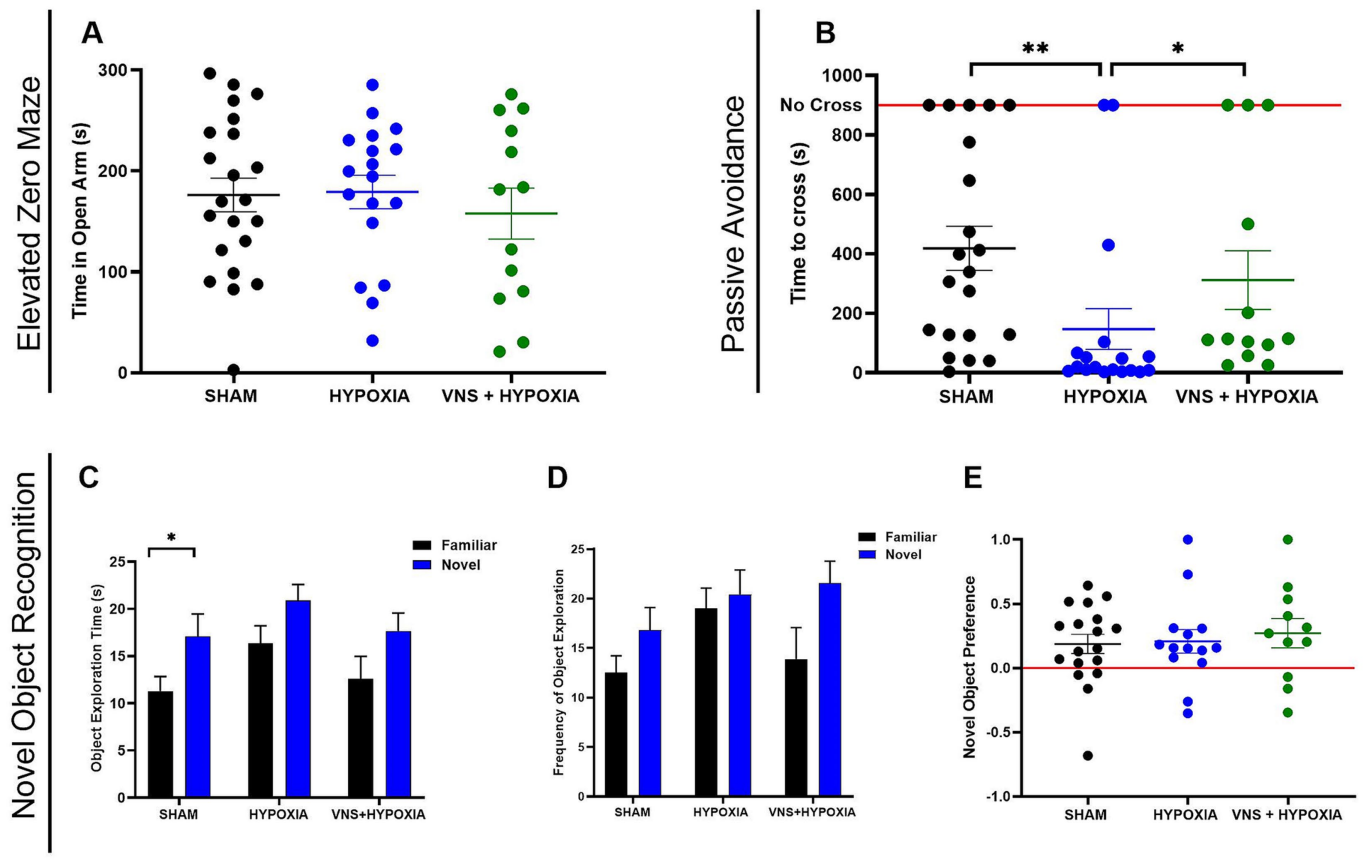
VNS mitigates hypoxia induced cognitive impairment in male rats. **(A)** The time spent in the open arm was not different for male rats in the sham, hypoxia, or VNS + hypoxia groups. **(B)** Hypoxia significantly decreased the latency to cross into the dark chamber compared to the sham rats by post-hoc analysis. VNS significantly increased the time to cross as compared to rats exposed to hypoxia based on post-hoc analysis. **(C)** In the NOR test, a two-way ANOVA found a significant effect of “object” for object exploration time, with post-hoc analysis showing this effect to be significant within the SHAM group. **(D)** A two-way ANOVA found a significant effect of ‘Object’ for frequency of object exploration, though post-hoc analysis did not find this effect to be significant within the groups. **(E)** Novel Object Preference was not significantly different among the groups. Data are presented as the mean ± SEM; *p < 0.05, **p < 0.01.

In our previous study, VNS paired with training enhanced performance in the NOR and PAT tests in male rats (Olsen et al., 2022). The same VNS intensity parameters were extended to this study to determine if VNS could augment performance deficits associated with hypoxia. To confirm the hypoxia exposure conditions used in this experimental design caused decreased performance, we paired exposure with behavioral paradigms to evaluate anxiety or cognition. Rats were exposed to four hours of hypoxic conditions with or without VNS intervention on training day, and the testing sessions were completed within 24 h of exposure (Figure 1). During the EZM testing session, anxiety-like behaviors were assessed by measuring the time spent in the open arm. There were no significant differences in time spent in the open arm among the sham (*n* = 22), hypoxia (*n* = 18), or VNS + hypoxia groups (*n* = 13), indicating hypoxia exposure with or without VNS did not affect anxiety-like behaviors (main effect *p* = 0.73, Figure 2A).

To determine the effects of hypoxia and VNS on recognition memory in the sham (*n* = 18), hypoxia (*n* = 14), and VNS + hypoxia (*n* = 11) groups, the NOR paradigm was performed. A two-way ANOVA analysis revealed a significant main effect of object for both object exploration time (*p* = 0.001, Figure 2C) and frequency of object exploration across all groups (*p* = 0.11, Figure 2D). However, when post-hoc analysis was conducted, a significant difference in exploration time between the familiar and novel objects was only observed within the sham group (*p* = 0.01). No significant differences were found in the frequency of object exploration between any groups. When normalized for total exploration time, there was no significant change in the NP between any of the groups (*p* = 0.81, Figure 2E). These results suggest hypoxia exposure alone or with VNS did not significantly affect recognition memory in male rats with this experimental study design.

To confirm the hypoxia exposure conditions used in this experiment caused decreased learning and memory performance, we assessed the effects of hypoxia or VNS intervention using the PAT. The testing session was completed 24 h after hypoxic exposure with or without VNS intervention. A significant difference in PAT performance, as assessed by the latency to cross into the dark arena, was detected among the sham (*n* = 21), hypoxia (*n* = 18), and VNS + hypoxia (*n* = 13) groups (main effect *p* = 0.001). Post-hoc analysis identified hypoxia exposure significantly reduced the PAT performance compared to the sham rats (*p* = 0.001, Figure 2B). As the hypoxia parameters decreased cognitive performance, we determined if VNS could restore this learning and memory deficit. VNS administered during hypoxia exposure significantly increased the PAT performance compared to the hypoxia group (*p* = 0.047). No significant difference in performance was observed between the sham group and the hypoxia-exposed stimulated group (*p* = 0.99) indicating VNS is sufficient to restore learning and memory deficits caused by hypoxia exposure in the PAT. This finding that VNS could augment hypoxia-induced impairments in an aversion learning and memory task led us to investigate the underlying mechanisms of hypoxia exposure and VNS intervention.

**Figure 3:**
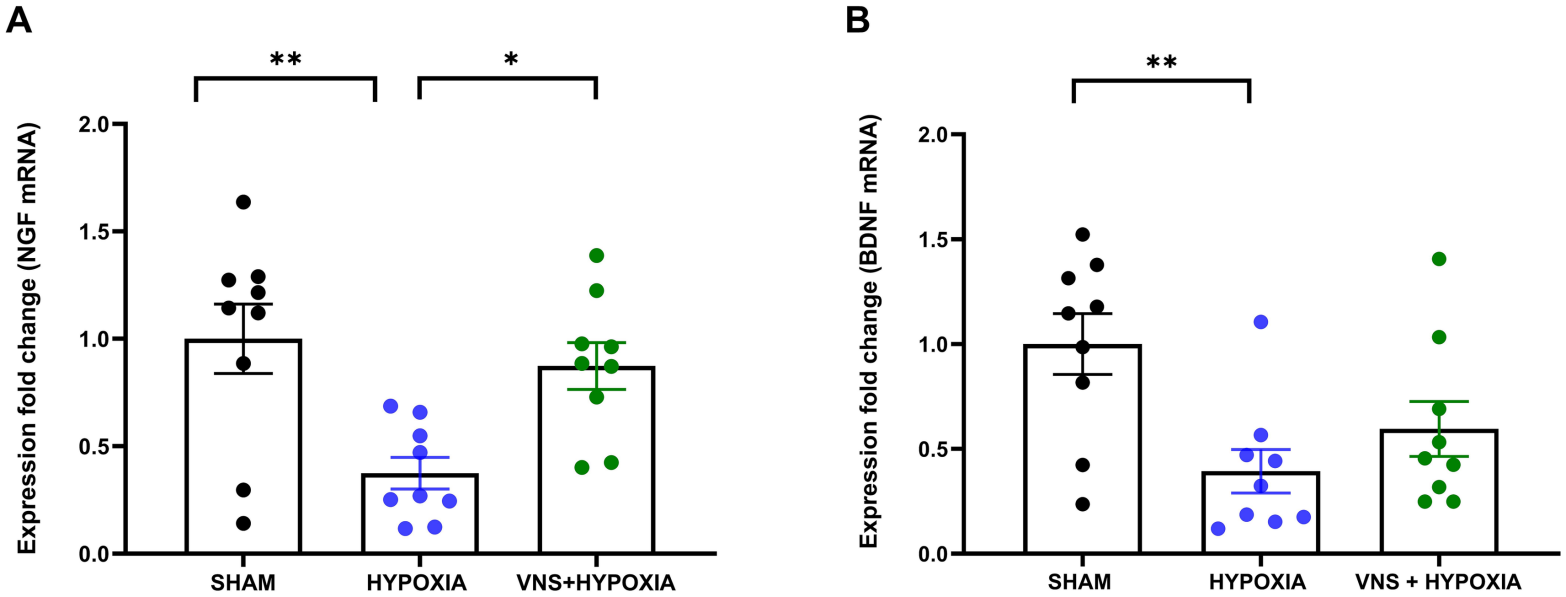
Hypoxia Decreases NGF and BDNF mRNA Expression in Hippocampus; VNS Restores Only NGF mRNA expression. **(A)** Post-hoc analysis identified NGF mRNA expression was significantly reduced between the sham and hypoxia groups. A significant increase in NGF mRNA between the hypoxia and VNS + hypoxia group was also identified by post-hoc analysis. **(B)** BDNF mRNA expression was significantly decreased with hypoxia exposure based on post-hoc analysis. Each bar graph represents the mean ± SEM; *p < 0.05 **p < 0.01.

To examine the effects of hypoxia and VNS, the expression levels of NGF and BDNF in the hippocampus, the brain region implicated in learning and memory, were examined. The hippocampus was macro dissected approximately 24 h after the last hypoxia exposure and VNS session to first analyze the selected neurotrophins by RT-qPCR. There was a significant difference in NGF mRNA expression among the three groups (*n* = 9 for sham, hypoxia, and VNS + hypoxia; main effect *p* = 0.003). *Post hoc* analysis revealed hypoxia exposure significantly decreased NGF mRNA expression compared to the sham rats (*p* = 0.004, Figure 3A). VNS intervention significantly increased NGF mRNA as compared to the hypoxia group (*p* = 0.02) and there was no significant difference with the sham group (*p* = 0.99). Similarly, there was a significant difference in BDNF expression among the three groups (*n* = 9 for sham, hypoxia, and VNS + hypoxia; main effect *p* = 0.008). Post hoc results showed significantly lower BDNF mRNA expression between the hypoxia group and sham group (*p* = 0.008, Figure 3B). While stimulation during hypoxic conditions caused a trending increase in BDNF expression as compared to the hypoxia exposure group, there was no significant difference (*p* = 0.82), indicating these stimulation parameters were not sufficient to restore the BDNF mRNA deficit caused by hypoxia.

**Figure 4:**
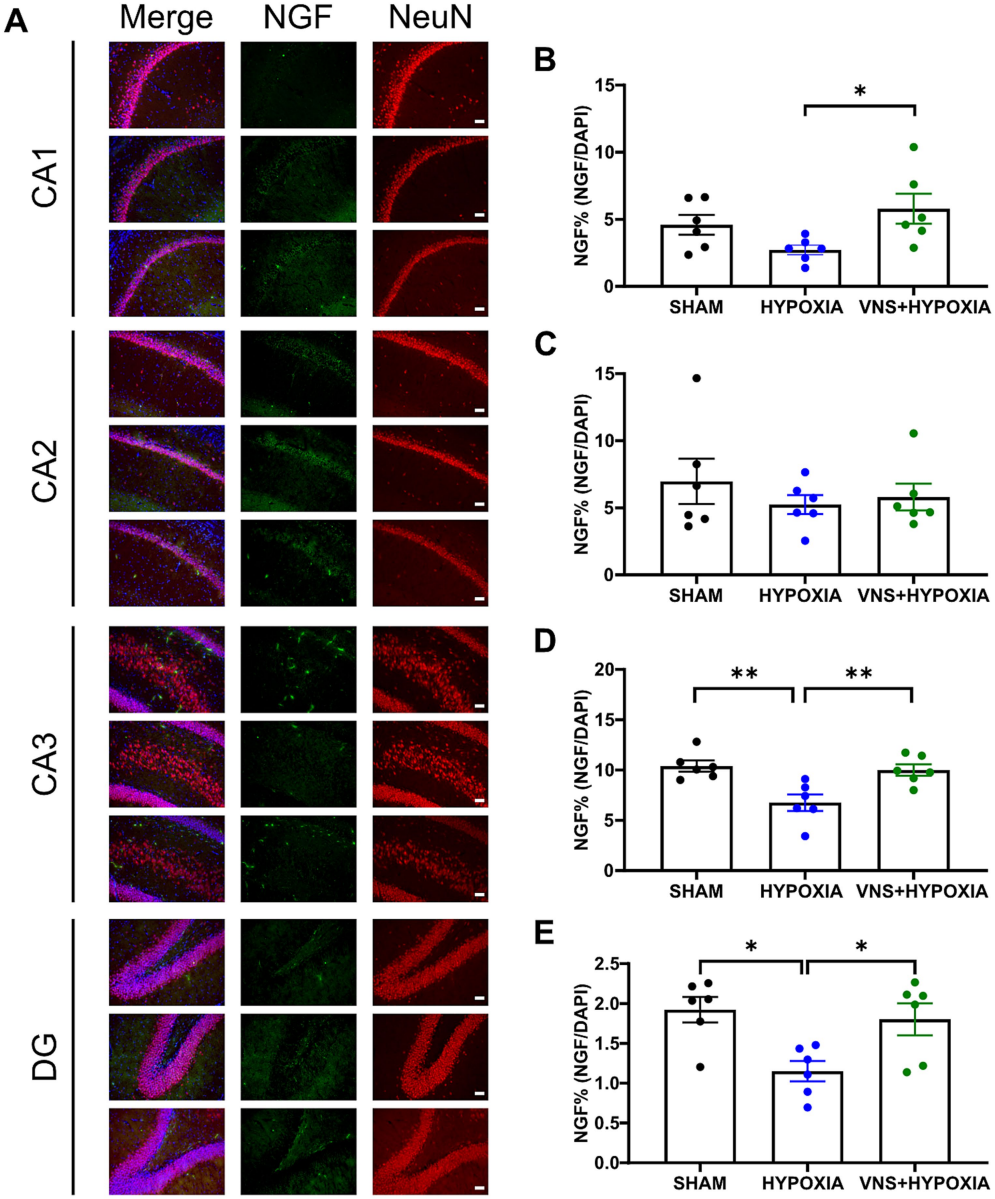
VNS mitigates hypoxia-induced reduction in NGF protein expression in the hippocampus. **(A)** Hypoxia and VNS augmented NGF protein expression in different regions of the hippocampus. **(B)** In the CA1 region, VNS significantly increased NGF protein expression compared to the hypoxia group as identified by post-hoc analysis. **(C)** In the CA2 region, no significant changes in NGF protein expression were detected between groups by one-way ANOVA. **(D)** Post-hoc analysis identified a significant reduction in NGF protein levels during hypoxia exposure when compared to the sham or VNS + hypoxia groups in the CA3 region. **(E)** Post-hoc analysis identified a significant reduction in NGF protein expression during hypoxia exposure when compared to the sham or VNS + hypoxia groups in the DG. Each bar graph represents the mean ± SEM; *p < 0.05, **p < 0.01. Scale bar = 50 μm.

To better understand if these changes in mRNA expression resulted in changes at the functional level, subregion-specific changes in NGF and BDNF protein expression levels in the hippocampus were investigated (*n* = 6 for sham, hypoxia, and VNS + hypoxia for NGF; *n* = 8 for sham, hypoxia, and VNS + hypoxia for BDNF). Hypoxia exposure effected the relative expression of NGF immunopositive cells in multiple hippocampal subfields (Figure 4A). A significant difference in NGF expression was found by one-way ANOVA among the sham, hypoxia, and VNS + hypoxia groups in the CA1 pyramidal layer (*p* = 0.049, Figure 4B), the CA3 stratum radiatum (*p* = 0.002, Figure 4D), and the DG subgranular zone (*p* = 0.01, Figure 4E). *Post hoc* analysis showed hypoxia significantly decreased NGF protein levels compared to the sham rats in the CA3 stratum radiatum and the DG subgranular zone (*p* = 0.004 and *p* = 0.015, respectively). Additionally, stimulation during hypoxia exposure significantly increased the NGF protein levels compared to the hypoxia group in the CA1 pyramidal layer, the CA3 stratum radatum, and the DG subgranular zone (*p* = 0.049, *p* = 0.01, and *p* = 0.04, respectively). In the CA2, no significant differences in NGF immunopositive cells were found between the sham, hypoxia, or VNS + hypoxia groups (*p* = 0.059, Figure 4C). Comparison of BDNF immunopositive cells between the sham, hypoxia, and VNS + hypoxia groups did not identify any significant changes in BDNF protein expression for any of the examined hippocampal regions (*p* > 0.05, Supplementary Figure 1).

## 4 Discussion

This study explored the effect of hypoxia exposure on performance and assessed whether VNS could mitigate impairments induced by hypoxia in healthy male rats. Hypoxia exposure decreased performance in the PAT and administering VNS during hypoxia successfully restored this cognitive performance deficit. Mechanistic analysis found hypoxia caused a decrease in NGF mRNA and protein expression in the hippocampus. VNS during hypoxia restored NGF mRNA expression in the hippocampus with NGF protein levels restored in the CA1, CA3, and DG.

In this study, rats were exposed to 8% oxygen to stimulate high altitude conditions of ~20,000 feet or higher, replicating the reduced oxygen availability encountered in high-altitude operations, such as military missions and aviation environments (Shaw et al., 2023). Our hypoxia conditions were not sufficient to induce notable changes in anxiety-like behavior or recognition memory as assessed by the EZM or NOR paradigms, respectively. Previous studies have shown an increase in anxiety-like behavior or a decrease in recognition memory in male rodent models (Kumari et al., 2020; Li et al., 2022). These behavioral changes occurred after significantly longer hypoxia exposure periods (i.e., days) suggesting the short hypoxia exposures (i.e., hours) used in this study are not sufficient to augment performance in these behavioral tests. Additionally, other hypoxia rodent studies utilized a NOR paradigm with only one hour between training and testing, and exposed animals to hypoxic conditions on the testing day (Zhu et al., 2023; Bekker et al., 2007). It is possible that hypoxia exposure in the present study was insufficient to induce an impairment in NOR performance because only the training session was targeted for hypoxia exposure. Incorporating additional tasks such as the Y-maze, Morris water maze, or Barnes maze, alongside varied hypoxia durations and testing timepoints, could provide a more complete assessment. Moreover, modifying behavioral test schedules and hypoxia exposure time may help uncover effects on anxiety like behavior and recognition memory that were not detected under the current conditions.

Four hours of exposure at 8% oxygen was sufficient to impair aversion learning and memory, which aligns with existing research in which three hours of exposure to hypobaric hypoxia significantly impacted PAT performance (Rybnikova et al., 2005). The decline in the PAT performance from hypoxia exposure was restored with VNS, indicating its potential to mitigate the adverse effects of hypoxia on learning and memory. Our study employed VNS during hypoxia exposure immediately before and after the behavioral training sessions to specifically target key periods of both memory acquisition and consolidation. This approach builds upon our previous work, where a single 30 min session of VNS after training significantly improved PAT performance in healthy male rats (Olsen et al., 2022). Moreover, in humans it has been shown that VNS applied before and after cognitive tasks can improve performance under stress conditions, such as sleep deprivation (McIntire et al., 2021). Translating stimulation protocols from rodents to humans remains challenging though. Most human studies use non-invasive stimulation methods that differ in current delivery and neural target engagement compared to implanted VNS in rodent models. Additionally, a limited number of studies have evaluated the cognitive effects of VNS in healthy individuals, and optimal parameters—including intensity, timing, and duration—are yet to be established (Olsen et al., 2023). While this study only examined hypoxia + VNS in healthy male rodents, there is also limited data on the effects of hypoxia in females. It also remains unclear how the estrous cycle interacts with hypoxia-induced cognitive changes and VNS mechanisms. Future work should include female subjects and consider hormonal cycling to elucidate sex-specific effects and improve translational relevance.

VNS is proposed to stimulate noradrenergic neurons in the LC, which leads to the release of NE in the hippocampus, facilitating memory consolidation and synaptic plasticity (Follesa et al., 2007; Chen et al., 2007; Hell et al., 1995; Tully and Bolshakov, 2010; Izumi and Zorumski, 1999). Previous research has shown that rats exposed to 10% oxygen for two hours had significantly reduced NE concentrations and turnover rate in the hippocampus when measured 24 h post-exposure (Miwa et al., 1986). Reduced NE turnover has also been linked to cognitive decline in stress and hypoxia studies (Banderet and Lieberman, 1989; Deijen and Orlebeke, 1994) with NE concentration regulating BDNF and NGF expression (Bathina and Das, 2015; Chen et al., 2007; Aloe et al., 2015; Mandela and Ordway, 2006). As the expression of NGF and BDNF have been shown to significantly increase after a single session of VNS in the hippocampus (Olsen et al., 2022; Rosso et al., 2019), we therefore examined their expression to investigate the effects of VNS on synaptic plasticity during hypoxia exposure.

Hypoxia exposure significantly reduced the expression of NGF mRNA in the hippocampus. To our knowledge, this is the first study to detect decreased NGF mRNA expression in the hippocampus after four hours of hypoxia exposure *in vivo*. VNS during hypoxia exposure may contribute to restored cognitive performance as stimulation increased NGF mRNA levels compared to the hypoxia group. These findings align with prior results which indicated VNS enhanced NGF mRNA expression in the hippocampus three days post-stimulation (Rosso et al., 2019). A translational reduction in NGF protein expression was also observed in the hippocampus with significant decreases localized to the CA3 and DG regions of hypoxia rats one day post hypoxia exposure. Previous work has also demonstrated regional specificity of hypoxia-induced synaptic changes with alterations in CA3 and DG neurons and network activities after one hour of hypoxia at 16% oxygen (Hencz et al., 2023). VNS administration during hypoxia exposure maintained NGF protein expression levels comparable to the sham group, underscoring its potential to address hypoxic damage. This effect could be attributed to increased synaptic transmission in the trisynaptic (DG-CA3-CA1) loop of the hippocampus via the activation of *β*-adrenergic receptors by VNS (Rocco et al., 2018; Olsen et al., 2023). NGF levels in the CA2 were not affected by hypoxia, likely due to unique cellular signaling pathways that render the CA2 highly resistant to stress and injury (Dudek et al., 2016). Future experiments aimed at further understanding the mechanisms of NGF in the hippocampus, especially under conditions that challenge normal functioning, could aid in the development of more effective strategies to restore cognitive function.

Hypoxia exposure significantly reduced the expression of BDNF mRNA in the hippocampus, consistent with *in vitro* findings showing decreased BDNF mRNA expression after three hours of hypoxia exposure (Tao et al., 2022). However, the stimulation conditions used in this study during hypoxia exposure did not restore BDNF mRNA expression back to sham levels. Although VNS was previously found to increase hippocampal BDNF, this effect may be limited to healthy subjects and not extend to the context of hypoxic stress (Follesa et al., 2007; Sanders et al., 2019). It is possible that within the context of hypoxia, differential modulation of Hypoxia-Induced Factor 1-alpha (HIF-1*α*) activity from hypoxia exposure could further modulate BDNF expression. Hypoxia has been shown to increase HIF-1α, which can bind to hypoxia-response elements on gene promoters, including promoter IV of the BDNF gene, leading to reduced BDNF mRNA expression (Sakata and Duke, 2014). Therefore, VNS-induced increases in BDNF via enhanced NE or serotonergic signaling may be dampened by HIF1-α downregulation of BDNF (Shin et al., 2019). Further research is needed to better understand the relationship between critical hypoxia pathways/factors and VNS.

The protein levels of BDNF remained unchanged in the hippocampus after hypoxia exposure. While a reduction in BDNF protein expression has been reported in hippocampal neuronal cultures after hypoxia exposure (5% oxygen) (Tao et al., 2022), it is challenging to directly compare tissue/cell oxygen exposure levels between *in vitro* and *in vivo* studies. BDNF expression was also examined three hours after hypoxia exposure for the *in vitro* study, whereas this study examined protein expression 24 h after exposure. Adlard and Cotman (2004) reported BDNF protein levels decrease at 5 and 10 h after stress but normalize by 24 h, indicating a transient suppression (Adlard and Cotman, 2004). It is possible that in our model, BDNF protein levels recovered by the time of assessment. In addition, post-transcriptional mechanisms, such as decreased ubiquitination, could enhance BDNF protein stability even when mRNA levels are low (Jia et al., 2008). The lack of effect of VNS on BDNF protein levels may reflect a limited influence of VNS on post-translational processes. Future research aimed at assessing whether VNS, including different stimulation parameters, can modulate degradation pathways could reveal more effective VNS protocols to counteract hypoxia-induced molecular changes in BDNF expression. Furthermore, neuronal death and apoptosis were not evaluated in this study, despite their relevance to hypoxic injury (Wang et al., 2022; Chen et al., 2017). Future experiments should examine apoptotic markers such as Bcl-2, Bax, and cleaved caspase-3 (Elmore, 2007) to determine whether VNS influences these pathways and promotes neuronal survival under hypoxic conditions.

This study provides insights into the effects of hypoxia exposure on cognition in male rats and demonstrates the effectiveness of VNS during hypoxia to augment performance. Specifically, we found four hours of hypoxia exposure can significantly impair aversion memory and learning. There were also significant reductions in NGF and BDNF expression in the hippocampus after exposure. Administration of VNS during hypoxia restored performance in the PAT and the expression of NGF. The VNS stimulation parameters utilized in this model have previously been confirmed to activate the VN and afferent pathways (Olsen et al., 2022). Therefore, the VNS effects found in this study may translate to human subjects using VNS devices that induce the same VN and afferent pathway activation. These findings enhance our understanding of the impact of hypoxia on neurotrophins expression and suggest VNS could be an effective strategy for countering hypoxia-induced cognitive impairments.

## Data Availability

The original contributions presented in the study are included in the article/Supplementary material, further inquiries can be directed to the corresponding author.
