# Supplementary material for: Vagus nerve stimulation ameliorates cognitive impairment caused by hypoxia
Source: Front Behav Neurosci. 2025 Jun 6;19:1555229. doi: 10.3389/fnbeh.2025.1555229 (PMC12179086; doi:10.3389/fnbeh.2025.1555229)
Supplement: Supplementary file 1 [file Table_1.docx]

Supplementary Material for

Vagus Nerve Stimulation Ameliorates Cognitive Impairment Caused by Hypoxia

Birendra Sharma^1,2^, Krysten A. Jones^1,3*^, Laura K. Olsen^1,2^, Raquel J. Moore^1,4^, Frances S. Curtner^1,4^ and Candice Hatcher-Solis^1^

^1^Neurobiology of Cognitive Performance, 711^th^ HPW, AFRL, Wright-Patterson AFB, OH

^2^Oak Ridge Institute for Science and Education, Oak Ridge, TN

^3^UES, Inc., BlueHalo Dayton, OH

^4^DCS Infoscitex, Dayton, OH

* Correspondence should be addressed to krysten.jones.ctr@us.af.mil

**Table of Contents**

Supplemental Table 1 S2

Supplemental Table 2 S2

Supplemental Table 3 S3

Supplemental Table 4 S3

Supplemental Table 5 S3

Supplemental Table 6 S4

Supplemental Table 7 S4

Supplemental Table 8 S4

Supplemental Figure 1 S5

| **Analysis** | **f** | **df** | **p-value** | **95% CI of difference** | **Mean±SEM** |
| --- | --- | --- | --- | --- | --- |
| *Elevated Zero Maze* | | | | | |
| Time in Open Arm | 0.315 | 2,50 | 0.731 | -141.7–210.9  -144.2–214.3  -102.8–212.9 | SHAM = 176.3 ± 16.64  HYPOXIA = 179.3 ± 16.61  VNS+HYPOXIA = 157.8 ± 25.27 |

**Supplemental Table 1**. Statistical analyses for EZM behavioral data.

| **Analysis** | **Kruskal-Wallis statistic** | **p-value** | **Mean ranks** | **95% CI of difference** | **Mean ± SEM** |
| --- | --- | --- | --- | --- | --- |
| *Passive Avoidance Test* | | | | | |
| Latency to cross | 13.30 | 0.001 | 33.45  16.22  29.50 | 264-573  2.43-291.9  96.19-526.7 | SHAM = 418.5 ± 74.06  HYPOXIA = 147.1 ± 68.59  VNS+HYPOXIA = 311.4 ± 98.80 |

**Supplemental Table 2**. Statistical analyses for PAT behavioral data.

| **Analysis** | **F** | **df** | **p-value** | **Mean ± SEM** |
| --- | --- | --- | --- | --- |
| *Novel Object Recognition- Object Exploration Time* | | | | |
| Main Effect Object | 9.664 | 1,38 | <0.001 | Familiar = 13.41 ± 1.52  Novel = 18.55 ± 1.12 |
| Main Effect Group | 6.281 | 2,38 | 0.11 | SHAM = 14.19 ± 1.48  HYPOXIA = 18.63 ± 1.30  VNS+HYPOXIA = 15.13 ± 1.61 |
| Interaction: Object and Group | 0.1195 | 2,38 | 0.931 | Familiar SHAM = 11.28 ± 1.55  Familiar HYPOXIA = 16.35 ± 1.86  Familiar VNS + HYPOIXA = 12.59 ± 2.39  Novel SHAM = 17.08 ± 2.37  Novel HYPOXIA = 20.91 ± 1.68  Novel VNS+HYPOXIA = 17.66 ± 1.91 |
| *Novel Object Recognition- Frequency of Object Exploration* | | | | |
| Main Effect Object | 6.052 | 2,38 | 0.112 | Familiar = 15.13 ± 1.98  Novel = 19.61 ± 1.42 |
| Main Effect Group | 6.575 | 2,38 | 0.011 | SHAM = 13.58 ± 1.2  HYPOXIA = 20.12 ± 1.73  VNS+HYPOXIA = 16.2 ± 2.19 |
| Interaction: Object and Group | 1.721 | 1,38 | 0.373 | Familiar SHAM = 12.5 ± 1.72  Familiar HYPOXIA = 19.0 ± 2.05  Familiar VNS+HYPOXIA = 13.88 ± 3.16  Novel SHAM = 16.83 ± 2.24  Novel HYPOXIA = 20.42 ± 2.46  Novel VNS+HYPOXIA = 21.56 ± 2.21 |

**Supplemental Table 3.** Statistical analyses for NOR behavioral data.

| **Analysis** | **f** | **df** | **p-value** | **95% CI of difference** | **Mean ± SEM** |
| --- | --- | --- | --- | --- | --- |
| *Novel Object Recognition* | | | | | |
| Novel Preference | 0.208 | 2,40 | 0.812 | 0.030-0.345  0.016-0.404  0.016-0.526 | SHAM = 0.1881 ± 0.0747  HYPOXIA = 0.2077 ± 0.09122  VNS+HYPOXIA = 0.0162 ± 0.5266 |

**Supplemental Table 4.** Statistical analyses for NOR NP behavioral data.

| **Analysis** | **f** | **df** | **p-value** | **95% CI of difference** | **Mean ± SEM** |
| --- | --- | --- | --- | --- | --- |
| *NGF mRNA Expression* | | | | | |
| Expression fold change | 7.542 | 2,24 | <0.01 | 0.6264-1.374  0.205-0.5441  0.6226-1.124 | SHAM = 1.000 ± 0.1620  HYPOXIA = 0.3745 ± 0.07354  VNS+HYPOXIA = 0.8735 ± 0.1088 |

**Supplemental Table 5**. Statistical analyses for Hippocampus NGF RT-qPCR data.

| **Analysis** | **f** | **df** | **p-value** | **95% CI of difference** | **Mean ± SEM** |
| --- | --- | --- | --- | --- | --- |
| *BDNF mRNA expression* | | | | | |
| Expression fold change | 5.854 | 2,24 | <0.01 | 0.6653-1.335  0.1544-0.6327  0.2936-0.8962 | SHAM = 1.000 ± 0.1451  HYPOXIA = 0.3936 ± 0.10377  VNS+HYPOXIA = 0.5949 ± 0.1307 |

**Supplemental Table 6**. Statistical analyses for Hippocampus BDNF RT-qPCR data.

| **Analysis** | **f** | **df** | **p-value** | **95% CI of difference** | **Mean ± SEM** |
| --- | --- | --- | --- | --- | --- |
| *NGF Expression* | | | | | |
| CA1  (Pyramidal region) | 3.711 | 2,15 | <0.05 | 2.693-6.490  1.793-3.653  2.912-8.657 | SHAM = 4.591 ± 0.7386  HYPOXIA = 2.723 ± 0.3618  VNS+HYPOXIA = 5.785 ± 1.117 |
| CA2  (Pyramidal region) | 0.536 | 2,15 | 0.596 | 2.613-11.32  3.410-7.061  3.238-8.362 | SHAM = 6.968 ± 1.694  HYPOXIA = 5.236 ± 0.7103  VNS+HYPOXIA = 5.800 ± 0.9966 |
| CA3  (Stratum radiatum) | 9.261 | 2,15 | <0.01 | 8.988-11.80  4.655-8.860  8.544-11.47 | SHAM = 10.40 ± 0.5478  HYPOXIA = 6.758 ± 0.8178  VNS+HYPOXIA = 10.01 ± 0.5685 |
| DG  (Subgranular zone) | 6.311 | 2,15 | 0.01 | 1.513-2.333  0.8238-1.478  1.286-2.320 | SHAM = 1.923 ± 0.1596  HYPOXIA = 1.151 ± 0.1272  VNS+HYPOXIA = 1.803 ± 0.2011 |

**Supplemental Table 7**. Statistical analyses for Hippocampus NGF IHC data.

| **Analysis** | **f** | **df** | **p-value** | **95% CI of difference** | **Mean ± SEM** |
| --- | --- | --- | --- | --- | --- |
| *BDNF Expression* | | | | | |
| CA1  (Pyramidal region) | 1.122 | 2,21 | 0.344 | 4.759-12.31  4.065-8.216  5.757-10.23 | SHAM = 8.534 ± 1.596  HYPOXIA = 6.140 ± 0.8777  VNS+HYPOXIA = 7.996 ± 0.9469 |
| CA2  (Pyramidal  Region) | 0.354 | 2,21 | 0.706 | 2.851-6.171  3.221-7.355  3.871-6.589 | SHAM = 4.511 ± 0.708  HYPOXIA = 5.288 ± 0.874  VNS+HYPOXIA = 5.230 ± 0.5746 |
| CA3  (Stratum radiatum) | 0.551 | 2,21 | 0.584 | 5.586-9.910  5.639-8.261  5.478-8.087 | SHAM = 7.748 ± 0.9144  HYPOXIA = 6.950 ± 0.5544  VNS+HYPOXIA = 6.783 ± 0.5516 |
| DG  (Subgranular zone) | 0.151 | 2,21 | 0.860 | 1.199-2.073  1.164-2.081  0.8129-2.838 | SHAM = 1.636 ± 0.1848  HYPOXIA= 1.622 ± 0.1940  VNS+HYPOXIA = 1.826 ± 0.4282 |

**Supplemental Table 8**. Statistical analyses for Hippocampus BDNF IHC data.


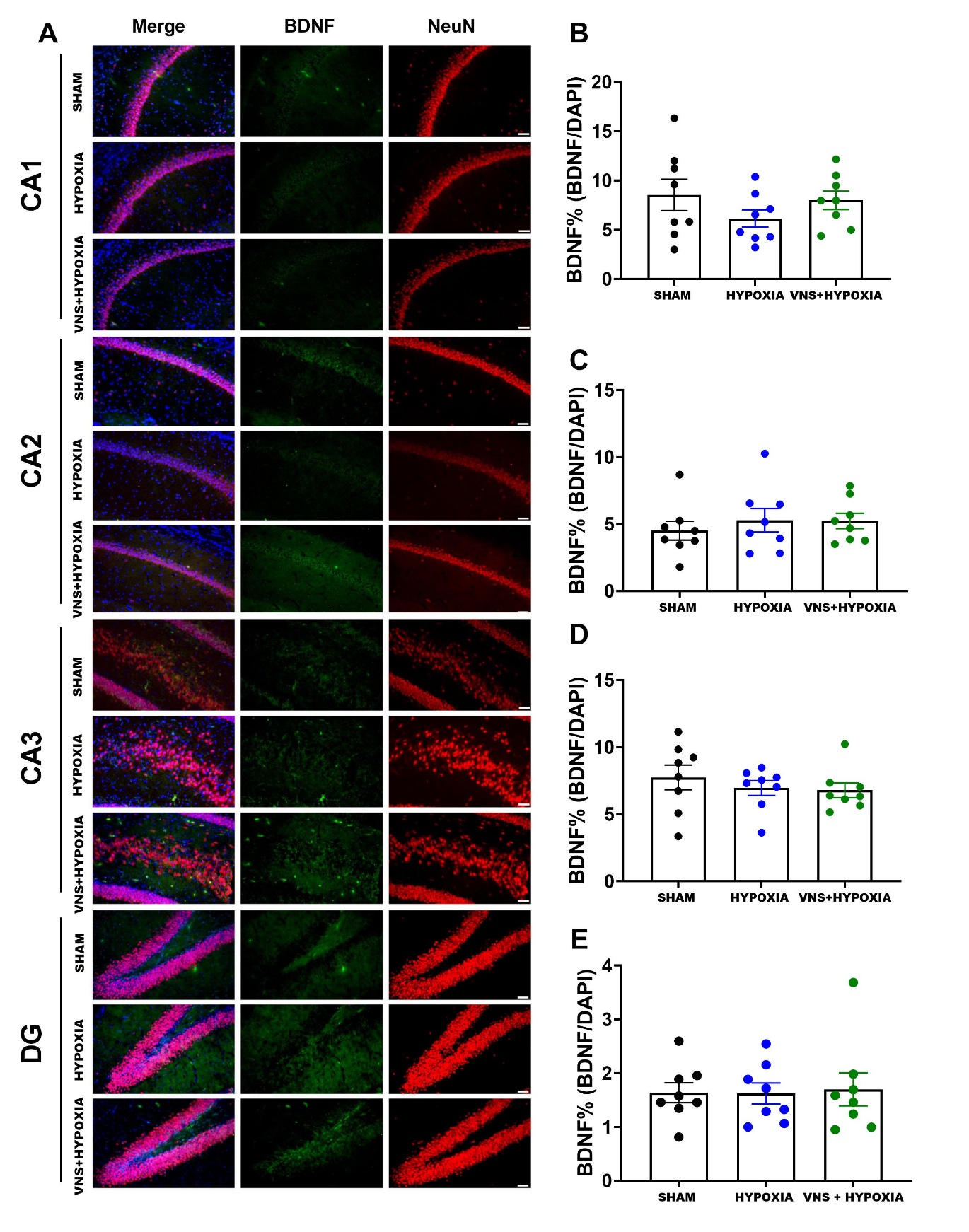


**Supplemental Figure 1. BDNF Protein Expression is Unaffected by Hypoxia or VNS + Hypoxia Across Hippocampal Regions**. Representative IHC images of BDNF expression (A). There were no significant changes in BDNF protein between the sham, hypoxia exposure, or VNS + hypoxia groups in the CA1, CA2, CA3, or DG regions of the hippocampus (B-E). Scale Bar = 50 µm. Error bars represent mean ± SEM.
